# Supplementary material for: MGMT genomic rearrangements contribute to chemotherapy resistance in gliomas
Source: Nat Commun. 2020 Aug 4;11:3883. doi: 10.1038/s41467-020-17717-0 (PMC7403430; doi:10.1038/s41467-020-17717-0)
Supplement: Supplementary file 1 — Supplementary Information [file 41467_2020_17717_MOESM1_ESM.pdf]

MGMT genomic rearrangements contribute to chemotherapy resistance in gliomas

Oldrini B et al.

Supplementary Information:

Supplementary Fig. 1-8

Supplementary Tables 1-4

Supplementary Data 1 is provided as separate Excel files

a

Category	Value
Total	252
Gender	
Male (%)	154 (61.1%)
Female (%)	98 (38.9%)
Median age (range)	45.5 (17-74)
Race	
Asian (%)	216 (85.7%)
Non-Asian (%)	36 (14.3%)
WHO Grade	
Grade II (%)	17 (6.7%)
Grade III (%)	38 (15.1%)
Grade IV (%)	197 (78.2%)
Molecular Subtype	
IDH wildtype	151 (59.9%)
IDH mutant, non-codel	71 (28.2%)
IDH mutant, codel	23 (9.1%)
Unknown	7 (2.8%)
Prior therapy	
Temozolomide (%)	252 (100%)

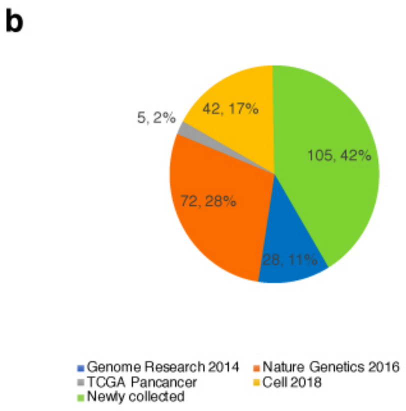

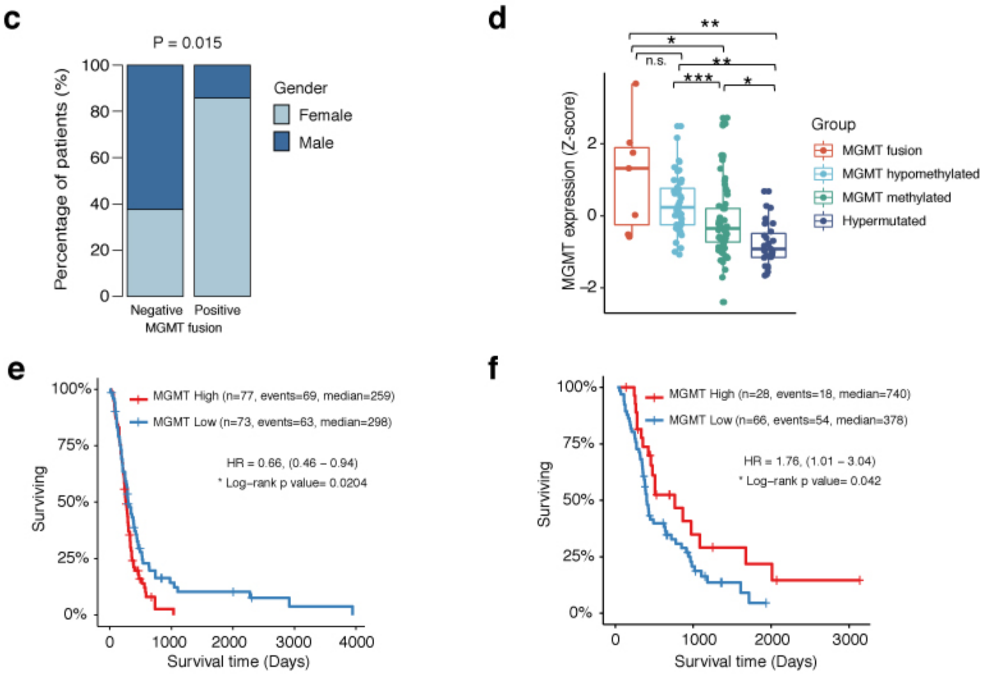

Supplementary Fig. 1 Further characterization of the study cohort and *MGMT* fusion**. a** Summary of the clinical features of the patients in this study. **b** Sources of the cases. 105 were newly collected, while 147 were from previous publications. **c** Gender distribution in MGMT fusion negative (153 males: 92 females) and positive (6 males: 1 female) glioma patients. The *P* value was calculated using two-sided Fisher's exact test. No multi-test correction was performed. **d** Comparison of MGMT expression level between patients with MGMT fusion (n=7), MGMT hypomethylation (n=49), MGMT methylation (n=63), and hypermutation (n=27). The bottom and top of each box represents the first and third quartiles, and the line inside is the median. The

whiskers correspond to 1.5 times the inter quartile range. *P* values were calculated by two-sided Wilcoxon rank-sum test. ***: P < 0.001; **: P <0.01; *: P < 0.001; n.s.: not significant. **e** Overall survival of IDH wildtype TMZ-treated recurrent glioma patients with high (n=77) and low (n=73) MGMT expression. The *P* value was calculated with Log-rank test. **f** Overall survival of IDH mutant TMZ-treated recurrent glioma patients with high (n=28) and low (n=66) MGMT expression. The *P* value was calculated with Log-rank test. Source data are provided as a Source Data file.

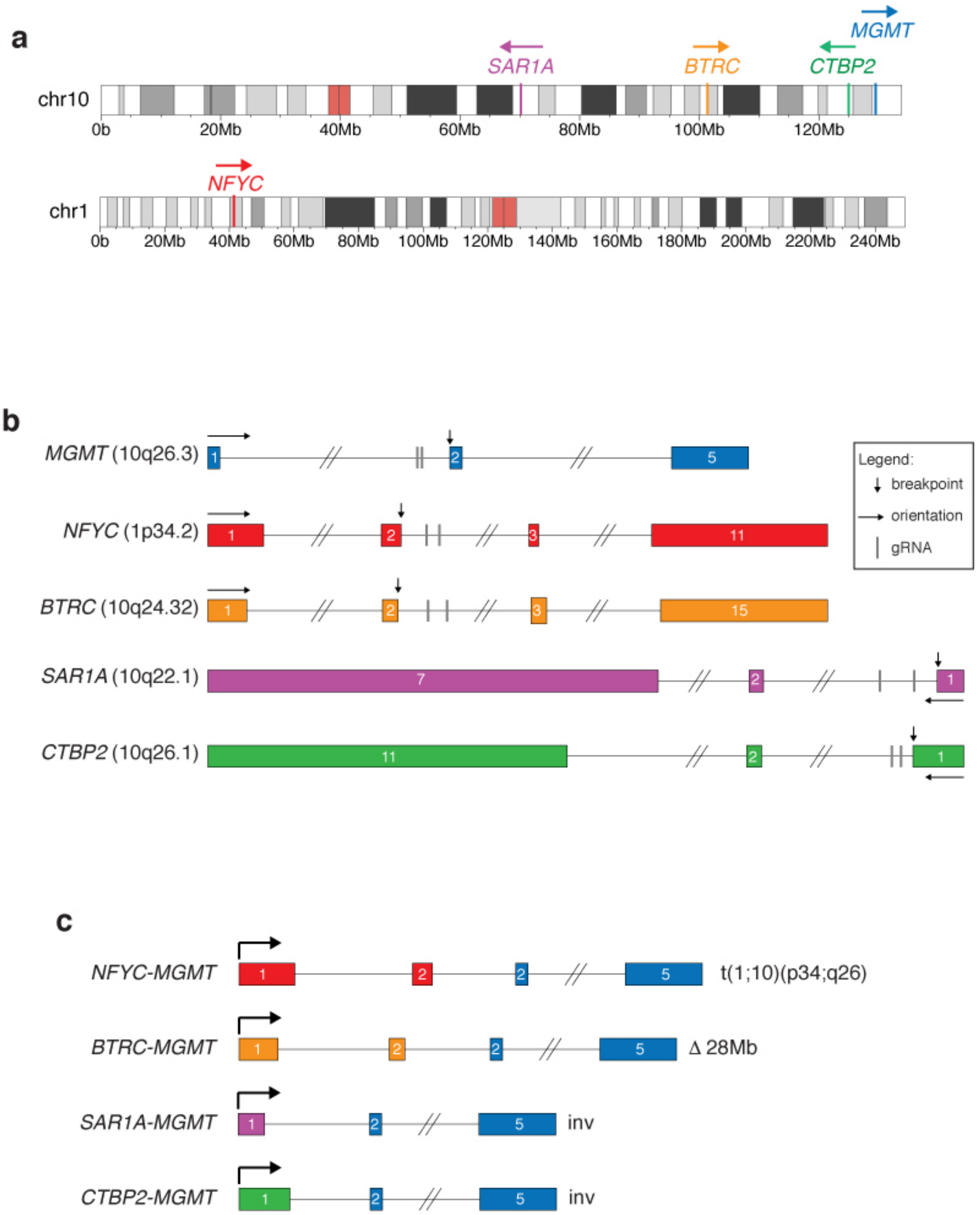

Supplementary Fig. 2 Generation of the *MGMT* fusions. **a** Chromosomal localization of *MGMT*, *SAR1A*, *BTRC*, *CTBP2* and *NFYC* genes. Arrow indicates gene orientation. **b** Schematic representation of the gene loci. Indicated are the breakpoints identified in patients, the gRNAs targeting the genes (Supplementary Table 4) and the gene orientations. **c** Schematic representation of the *MGMT* genomic rearrangements showing translocation, deletion and inversion events.

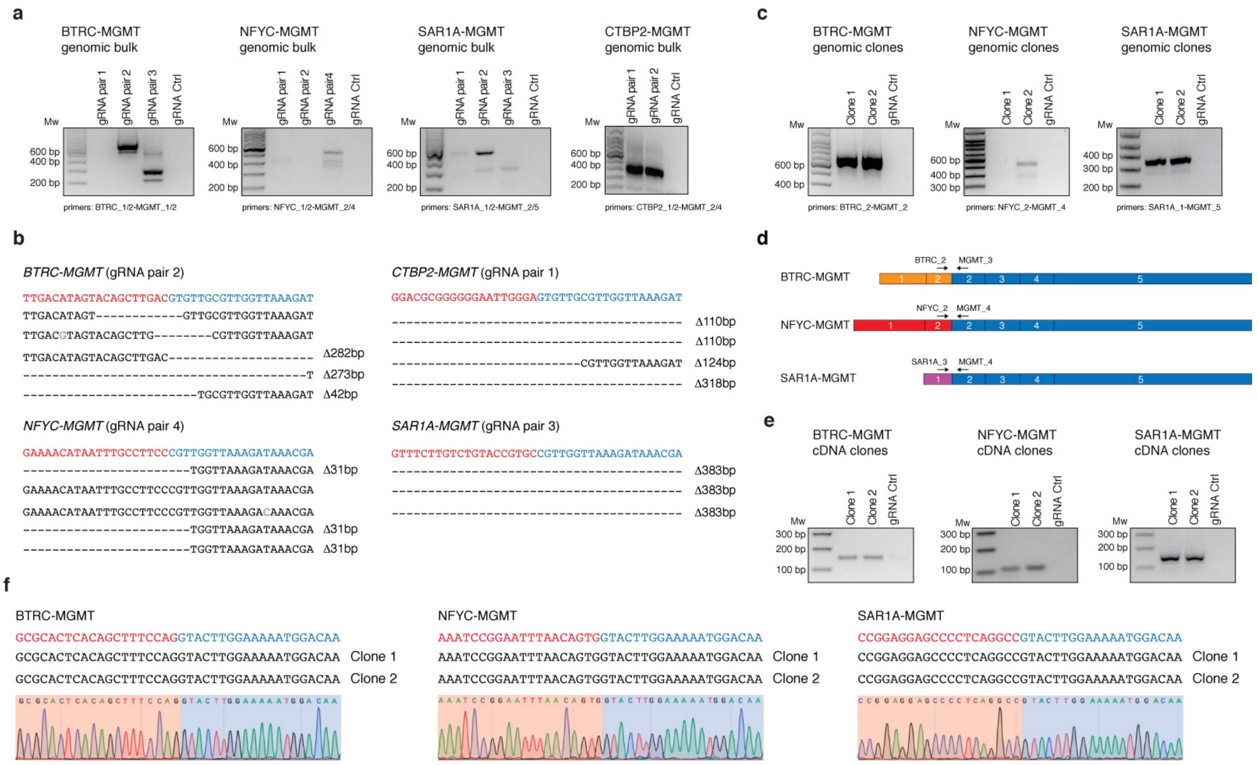

Supplementary Fig. 3 Validation of *MGMT* fusions. **a** Nested PCR analysis performed with the specific primers for each fusion on genomic DNA extracted from the U251 transduced with the indicated gRNA pairs (see Supplementary Table 5). **b** Sequence examples of the PCR products in (a). Deletions were found in most sequences and are displayed as dashes; nucleotides in green represent point mutations. **c** PCRs with specific primers performed on genomic DNA extracted from two independent TMZ-resistant clones per MGMT fusions. **d** Schematic representation of MGMT fusion transcripts with the primers used for the PCR amplification of the fusion regions (see Supplementary Table 3). **e** RT-PCRs with primers showed in (d) performed on mRNA extracted from two independent TMZ-resistant clones per *MGMT* fusions. **f** The PCR bands in (e) were sub-cloned and analyzed by Sanger sequencing. The sequences of the two independent clones and a representative chromatogram are shown.

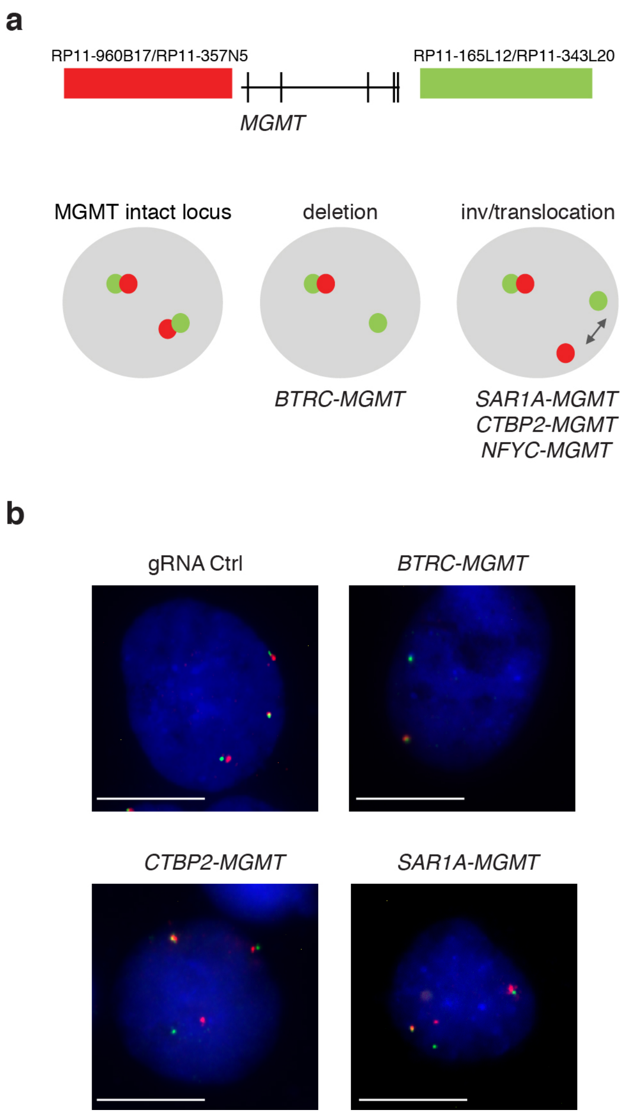

Supplementary Fig. 4. Break-apart fluorescence in situ hybridization (FISH) assay. **a** Schematic representation of *MGMT* locus with the BAC clones used for the FISH (*top panel*) and example of the expected results for the indicated rearrangements (*bottom panel*). **b** Representative FISH images of n=2 biologically independent experiments of U87 cells carrying the indicated MGMT rearrangement. Scale bar: 5 µm.

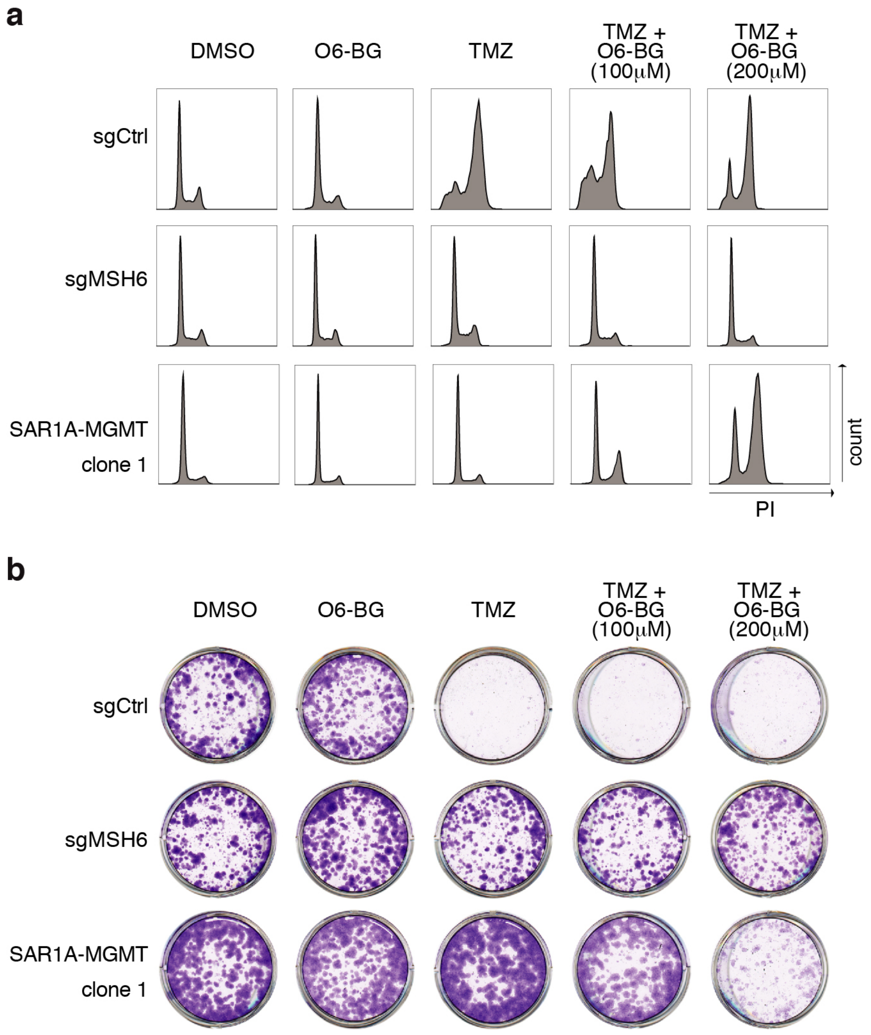

Supplementary Fig. 5 Increased doses of O6-BG significantly enhance TMZ cytotoxic effect in cells with high MGMT expression level. **a** Cell cycle distribution of U251 SAR1A-MGMT clone 1 in presence of O6-BG (100µM-200µM), TMZ (100µM) or combination of TMZ and O6-BG with the indicated doses for 72h, measured by propidium iodide (PI) staining and FACS. **b** Colony forming assay on U251 SAR1A-MGMT clone 1 grown for 12 days with the same drug concentration described in (a). U251 sgCtrl and sgMSH6 are used as controls for both experiments.

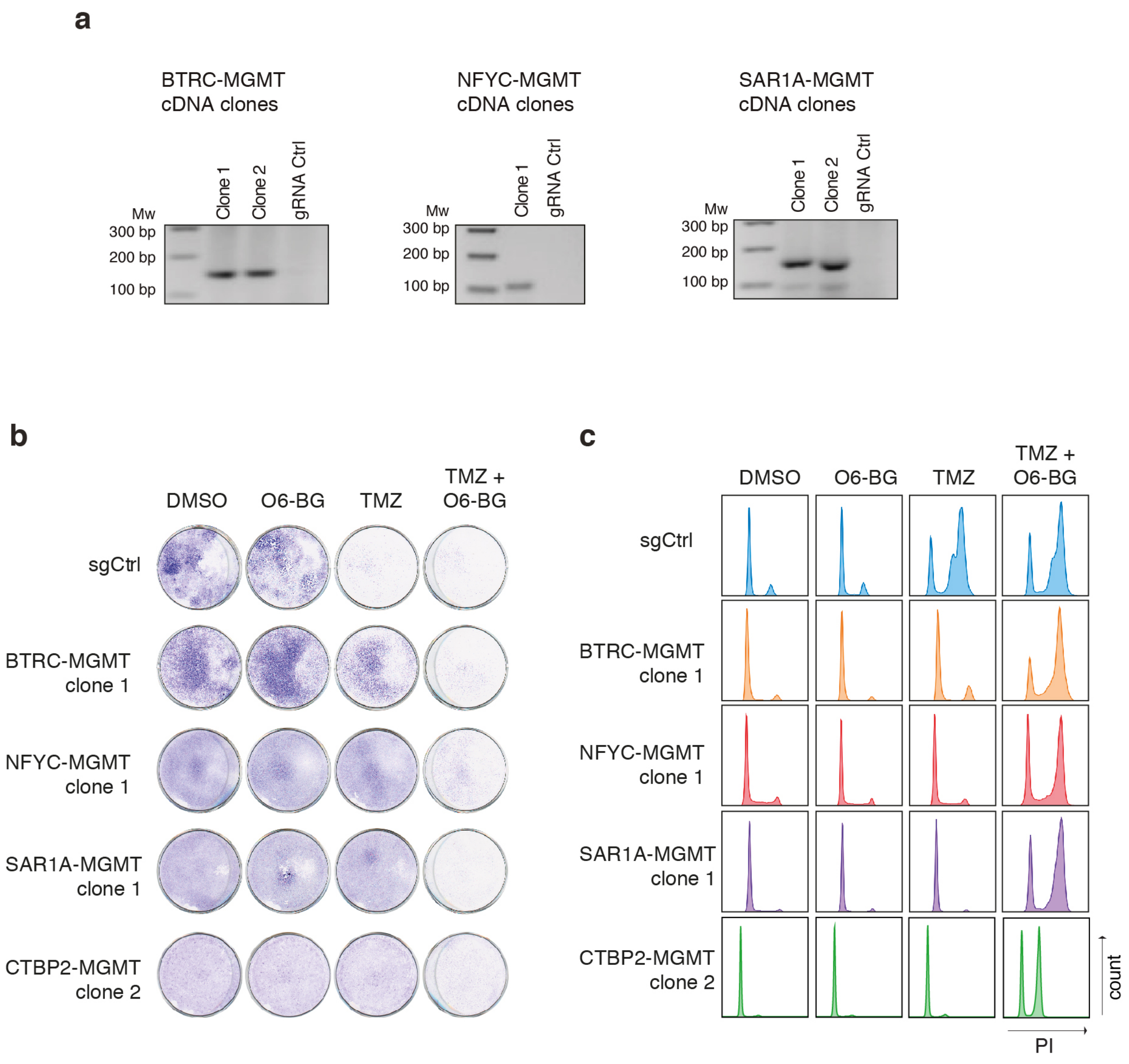

Supplementary Fig. 6. Validation of the *MGMT* fusions induced TMZ resistance in U87 GBM cell line. **a** PCR analysis performed with the specific primers (see Supplementary Fig. 3d) for each fusion on cDNA from TMZ resistant U87 clones. **b** Clonogenic assay of TMZ resistant U87 single cell clones expressing MGMT fusions compared to sgCtrl cells exposed to O_6-BG (100µM) or/and TMZ (100µM) for 12 days. **c** Cell cycle distribution of TMZ resistant U87 MGMT fusion clones compared to sgCtrl cells in presence of O_6-BG (100µM) or/and TMZ (100µM) for 72h, measured by propidium iodide (PI) staining and FACS.

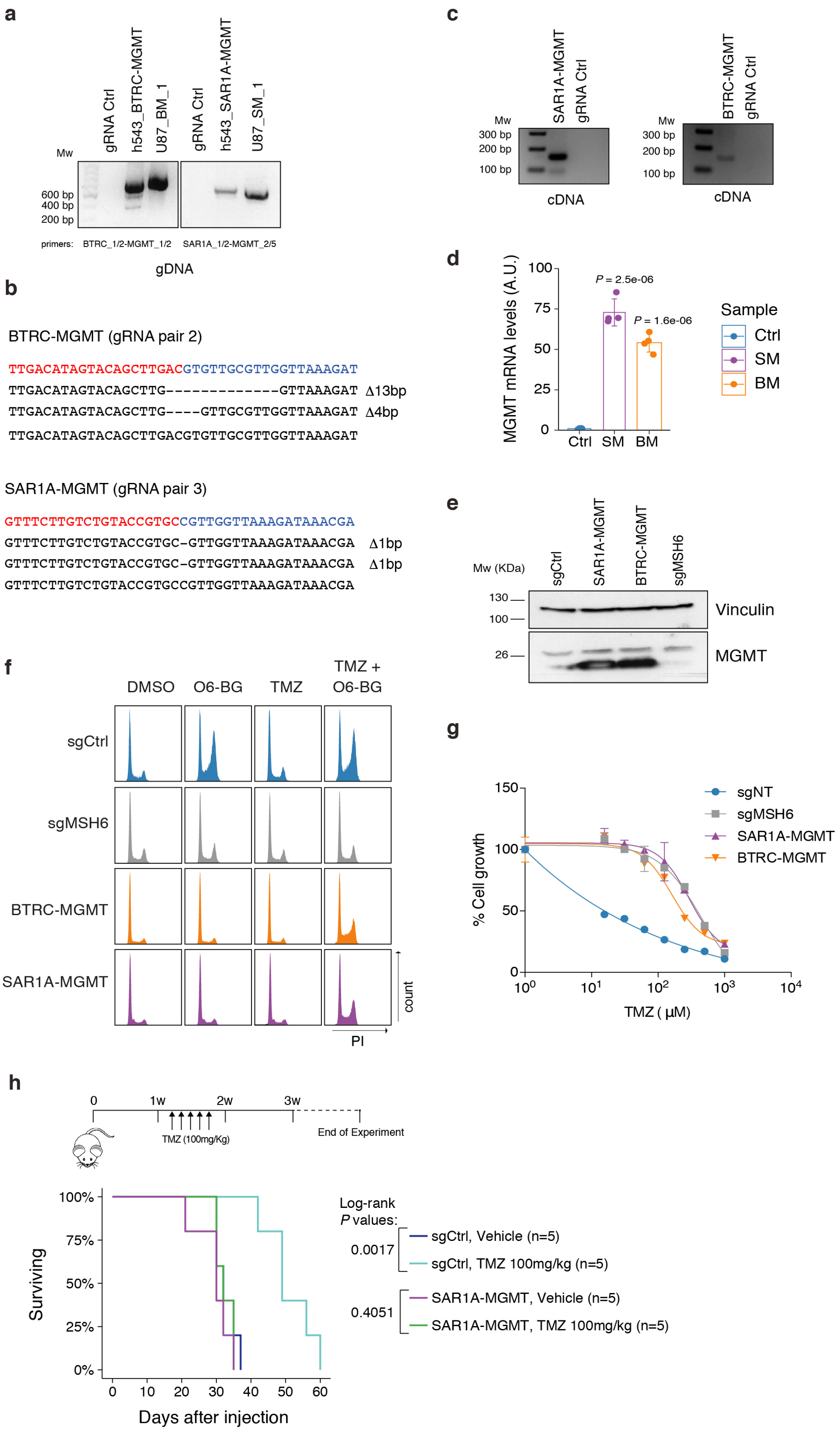
a
gRNA Ctrl
h543_BTRC-MGMT
U87_BM_1
gRNA Ctrl
h543_SAR1A-MGMT
U87_SM_1
Mw
600 bp
400 bp
200 bp
primers: BTRC_1/2-MGMT_1/2 SAR1A_1/2-MGMT_2/5
gDNA
b
BTRC-MGMT (gRNA pair 2)
TTGACATAGTACAGCTTGACGTGTTGCGTTGGTTAAAGAT
TTGACATAGTACAGCTTG-------------GTTAAAGAT Δ13bp
TTGACATAGTACAGCTTG----GTTGCGTTGGTTAAAGAT Δ4bp
TTGACATAGTACAGCTTGACGTGTTGCGTTGGTTAAAGAT
SAR1A-MGMT (gRNA pair 3)
GTTTCTTGTCTGTACCGTGCCGTTGGTTAAAGATAAACGA
GTTTCTTGTCTGTACCGTGC-GTTGGTTAAAGATAAACGA Δ1bp
GTTTCTTGTCTGTACCGTGC-GTTGGTTAAAGATAAACGA Δ1bp
GTTTCTTGTCTGTACCGTGCCGTTGGTTAAAGATAAACGA
c
SAR1A-MGMT
gRNA Ctrl
BTRC-MGMT
gRNA Ctrl
Mw
300 bp
200 bp
100 bp
cDNA
d
MGMT mRNA levels (A.U.)
100
75
50
25
0
Ctrl SM BM
P = 2.5e-06
P = 1.6e-06
Sample
Ctrl
SM
BM
e
Mw (KDa)
sgCtrl
SAR1A-MGMT
BTRC-MGMT
sgMSH6
130
100
26
Vinculin
MGMT
f
DMSO
O6-BG
TMZ
TMZ + O6-BG
sgCtrl
sgMSH6
BTRC-MGMT
SAR1A-MGMT
count
PI
g
% Cell growth
150
100
50
0
10^0 10^1 10^2 10^3 10^4
TMZ (μM)
sgNT
sgMSH6
SAR1A-MGMT
BTRC-MGMT
h
0
1w
2w
3w
TMZ (100mg/Kg)
End of Experiment
Surviving
100%
75%
50%
25%
0%
0 10 20 30 40 50 60
Days after injection
Log-rank P values:
0.0017
sgCtrl, Vehicle (n=5)
sgCtrl, TMZ 100mg/kg (n=5)
0.4051
SAR1A-MGMT, Vehicle (n=5)
SAR1A-MGMT, TMZ 100mg/kg (n=5)

Supplementary Fig. 7 Validation of the *MGMT* fusions induced TMZ resistance in patient derived tumor spheres. **a** Nested PCR analysis performed with the specific primers for each fusion on genomic DNA extracted from the h543 tumor spheres (see Supplementary Table 2). U87 clones expressing BTRC-MGMT and SAR1A-MGMT fusions used as controls. **b** Sequence examples of the PCR products in (a). **c** PCR analysis performed with the specific primers (see Supplementary Fig. 3d) for each fusion on cDNA. **d** MGMT quantitative-PCR performed on mRNA from h543 expressing the indicated MGMT fusions and control. Data are from a representative experiment of n=2 biological replicate. Centre of the bars represent the mean (technical replicate n=4) and the error bars are the standard deviations. Two sided Student's *t* test with Bonferroni adjustment for multiple comparisons: Ctrl vs SAR1A-MGMT *P* = 7.60e-6; Ctrl vs BTRC-MGMT *P* = 6.50e-6. **e** Western blot analysis of MGMT protein levels in h543 expressing the indicated *MGMT* fusions. h543 sgCtrl and sgMSH6 are used as controls. **f** Cell cycle distribution of H543 expressing BTRC-MGMT and SAR1A-MGMT fusions compared to sgCtrl and shMSH6 cells in presence of O_6-BG (100µM) or/and TMZ (100µM) for 72h, measured by propidium iodide (PI) staining and FACS. **g** Viability of h543 expressing BTRC-MGMT and SAR1A-MGMT fusions compared to sgCtrl and shMSH6 upon exposure to different concentrations of TMZ measured by MTT. Values are represented as percentage of cell viability compared to DMSO control. Data are from a representative experiment of n = 2 biologically independent experiments, and presented as mean (technical replicate n=4) and standard deviation. **h** *Top panel*: scheme of the *in vivo* experimental design. *Bottom panel*: Kaplan-Meier survival curve of animals intracranially injected with H543 sgCtrl and H543 SAR1A-MGMT tumor spheres treated or not with TMZ (100mg/Kg) for 5 days. n=5 animals per group. sgCtrl Log-rank *P* value = 0.0017, SAR1A-MGMT Log-rank *P* value = 0.4051. Source data are provided as a Source Data file.

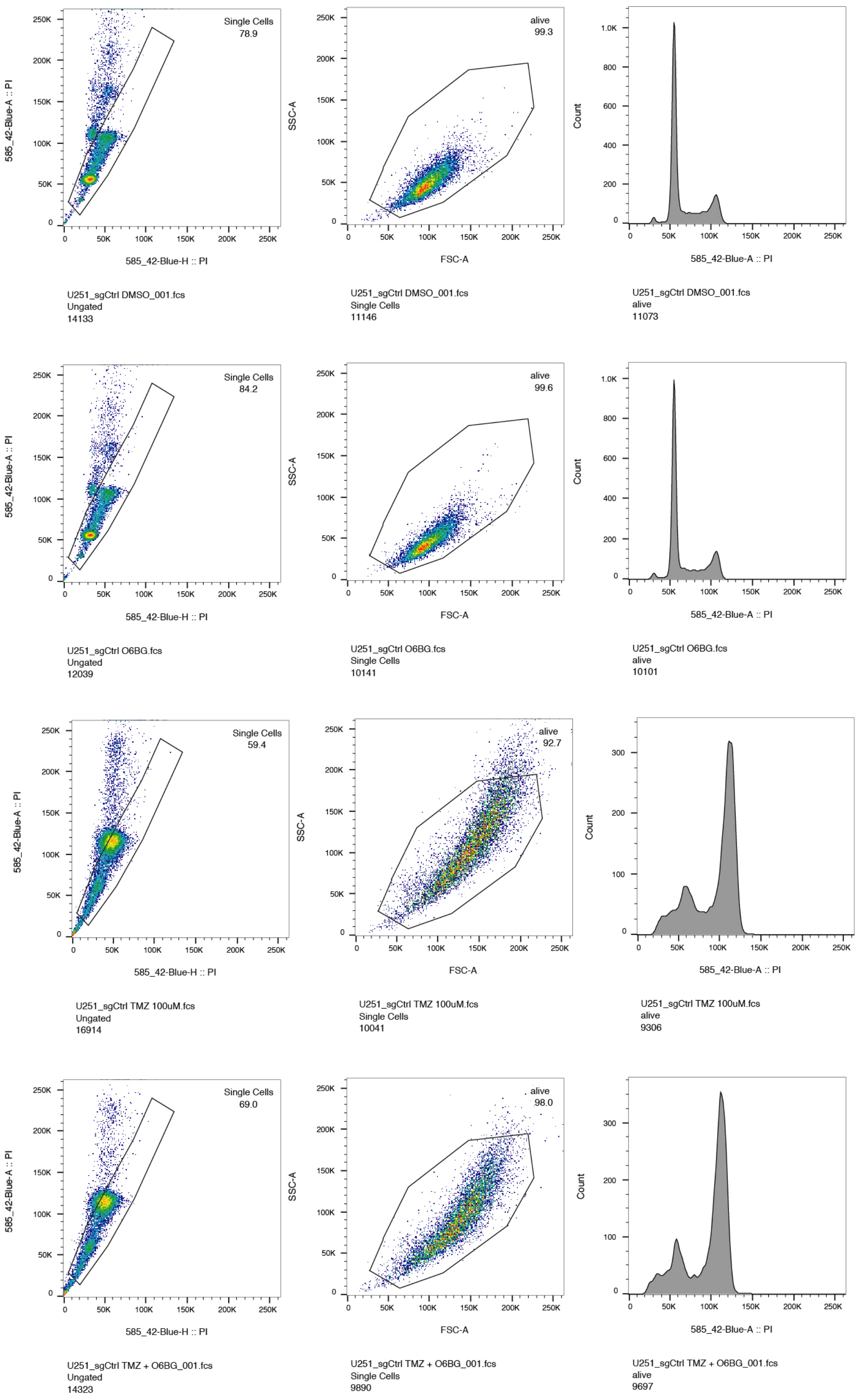

Supplementary Fig. 8 Examples of gating strategy used for the PI staining analysis. U251 sgCtrl samples from Figure 3b (top to bottom): DMSO, O6BG, TMZ and O6BG + TMZ.

Supplementary Table 1

Sample ID	Fusion Name	Left Gene	Left Breakpoint	Right Gene	Right Breakpoint	Junction Read Count	Spanning Fragment Count
CGGA_1199	GLRX3--MGMT	*GLRX3*	chr10:131943583:+	*MGMT*	chr10:131334505:+	2090	324
CGGA_1729	CAPZB--MGMT	*CAPZB*	chr1:19746155:-	*MGMT*	chr10:131334505:+	43	5
CGGA_P356	FAM175B--MGMT	*FAM175B*	chr10:126490470:+	*MGMT*	chr10:131334505:+	155	0
CGGA_1707	RPH3A--MGMT	*RPH3A*	chr12:113230068:+	*MGMT*	chr10:131334505:+	12	0
R114R	NFYC--MGMT	*NFYC*	chr1:41204620:+	*MGMT*	chr10:131334505:+	96	120
R114R	BTRC--MGMT	*BTRC*	chr10:103190209:+	*MGMT*	chr10:131334505:+	67	85
R056R	SAR1A--MGMT	*SAR1A*	chr10:71930169:-	*MGMT*	chr10:131334505:+	4	4
TCGA-FG-5965-02B	CTBP2--MGMT	*CTBP2*	chr10:126849396:-	*MGMT*	chr10:131334505:+	3	4

Supplementary Table 1. Breakpoint information of the identified MGMT fusions

Supplementary Table 2

Primer_name	Sequence (5'-3')	Gene	Application
BTRC_1	CCTGTAATCTGTGCCATCCTGT	BTRC	genomic
BTRC_2	TGCCTGTATAACCCAGGGAC	BTRC	cDNA
CTBP2_1	AGGAGCGAGGAGTGAGCG	CTBP2	genomic
CTBP2_2	GCAAATAGCCGGGAGGCGC	CTBP2	genomic
MGMT_1	CATGTGCGGTATACAGGATCACGTGG	MGMT	genomic
MGMT_2	CCCTTGCCCAGGAGCTTTATT	MGMT	genomic
MGMT_3	TTATTTCGTGCAGACCCTGCT	MGMT	cDNA
MGMT_4	GTCCAGTGTGGTGCGTTTCA	MGMT	genomic/cDNA
MGMT_5	CAGCATGTGCGGTATACAGGA	MGMT	genomic
NFYC_1	GTTGTCGAGATGTCCACAGAAGGAG	NFYC	genomic
NFYC_2	CCCAGCAAAGCCTACAGTCG	NFYC	cDNA
SAR1A_1	GTGAGTCTGAGGGTCGCGT	SAR1A	genomic
SAR1A_2	CGGAAGGCGGGGAGGTC	SAR1A	genomic
SAR1A_3	GACGTACATCCGGCGAGTAG	SAR1A	cDNA
FAM175B_MGMT	GGCTACACCTTCAGTGCTGT	FAM175B	cDNA
MGMT_FAM175B	CGGGGAACTCTTCGATAGCC	MGMT	cDNA
CAPZB_MGMT	CTGGACTGTGCCTTGGACCTA	CAPZB	cDNA
MGMT_CAPZB	GTCCTCCGGAGTAGTTGCC	MGMT	cDNA
F5	TTTGAGACTATAAATATGCATGCGAGAAAAGCCTTGTTTG	*NA*	Cloning
R1	GACTAGCCTTATTTTAACTTGCTATTTCTAGCTCTAAAAC	*NA*	Cloning
MGMT_qPCR_1F	GTGATTTCTTACCAGCAATTAGCA	MGMT	qPCR/ cDNA
MGMT_qPCR_1R	CTGCTGCAGACCACTCTGTG	MGMT	qPCR/cDNA
bACTIN_qPCR_F	CAAGGCCAACCGCGAGAAGAT	ACTIN	qPCR/cDNA
bACTIN_qPCR_R	CCAGAGGCGTACAGGGATAGCAC	ACTIN	qPCR/cDNA
Met_MGMT_Fw	TTTCGACGTTCGTAGGTTTTCGC	MGMT	MPS
Met_MGMT_Rv	GCACTCTTCCGAAAACGAAACG	MGMT	MPS
UnMet_MGMT_Fw	TTTGTGTTTTGATGTTTGTAGGTTTTTGT	MGMT	MPS
UnMet_MGMT_Rv	AACTCCACACTCTTCCAAAAACAAAACA	MGMT	MPS

Supplementary Table 2. Primers used in this study

Supplementary Table 3

Gene_name	gRNA_name	gRNA_sequence	PAM	Position (GRCh38)	Strand
MGMT	MGMT_gRNA1	TTTAACCAACGCAACACCGA	AGG	chr10:129535957-129535979	-1
MGMT	MGMT_gRNA2	AGCTTCCTTCGGTGTTGCGT	TGG	chr10:129535952-129535974	1
BTRC	BTRC_gRNA1	TACTGAGTGATGTGAGATAC	AGG	chr10:101430570-101430952	-1
BTRC	BTRC_gRNA2	ACATAGTACAGCTTGACTGT	TGG	chr10:101430675-101430697	1
NFYC	NFYC_gRNA1	AGTGTTCTTCCATAGGCGTG	TGG	chr1:40739146-40739168	1
NFYC	NFYC_gRNA2	AACATAATTTGCCTTCCGTG	AGG	chr1:40739098-40739120	1
SAR1A	SARA1_gRNA1	ATTTCGTAAACCGGAGCGCA	CGG	chr10:70170115-70170137	1
SAR1A	SARA1_gRNA2	TGACACGCAGGCGGTTCTGT	GGG	chr10:70170280-70170302	-1
CTBP2	CTBP2_gRNA1	CGCGGGGGGAATTGGGAACC	GGG	chr10:125160722-125160744	-1

Supplementary Table 3. gRNA used in this study

Supplementary Table 4

gRNA_pair_name	fusion	gRNA_A	gRNA_B
BTRC_MGMT_1	BTRC_MGMT	MGMT_gRNA1	BTRC_gRNA1
BTRC_MGMT_2	BTRC_MGMT	MGMT_gRNA1	BTRC_gRNA2
BTRC_MGMT_3	BTRC_MGMT	MGMT_gRNA2	BTRC_gRNA1
NFYC_MGMT_1	NFYC_MGMT	MGMT_gRNA1	NFYC_gRNA1
NFYC_MGMT_2	NFYC_MGMT	MGMT_gRNA1	NFYC_gRNA2
NFYC_MGMT_4	NFYC_MGMT	MGMT_gRNA2	NFYC_gRNA2
SAR1A_MGMT_1	SARA1_MGMT	MGMT_gRNA1	SARA1_gRNA1
SAR1A_MGMT_2	SARA1_MGMT	MGMT_gRNA1	SARA1_gRNA2
SAR1A_MGMT_3	SARA1_MGMT	MGMT_gRNA2	SARA1_gRNA1
CTBP2_MGMT_1	CTBP2_MGMT	MGMT_gRNA1	CTBP2_gRNA1
CTBP2_MGMT_2	CTBP2_MGMT	MGMT_gRNA1	CTBP2_gRNA2

Supplementary Table 4. gRNA pairs. See Supplementary Table 3 for gRNAs sequence
