# Supplementary material for: MGMT genomic rearrangements contribute to chemotherapy resistance in gliomas
Source: Nat Commun. 2020 Aug 4;11:3883. doi: 10.1038/s41467-020-17717-0 (PMC7403430; doi:10.1038/s41467-020-17717-0)
Supplement: Supplementary file 3 — Description of Additional Supplementary Files [file 41467_2020_17717_MOESM3_ESM.pdf]

**Description of Additional Supplementary Files**

File Name: Supplementary Data 1
Description: contains the clinical information of the cohort presented in this study.
