# Supplementary material for: MGMT genomic rearrangements contribute to chemotherapy resistance in gliomas
Source: Nat Commun. 2020 Aug 4;11:3883. doi: 10.1038/s41467-020-17717-0 (PMC7403430; doi:10.1038/s41467-020-17717-0)
Supplement: Supplementary file 5 — Reporting Summary [file 41467_2020_17717_MOESM5_ESM.pdf]

nature research

Corresponding author(s): Massimo Squatrito
Jiguang Wang
Tao Jiang

Last updated by author(s): June 10, 2020

Reporting Summary

Nature Research wishes to improve the reproducibility of the work that we publish. This form provides structure for consistency and transparency in reporting. For further information on Nature Research policies, see our Editorial Policies and the Editorial Policy Checklist.

Statistics

For all statistical analyses, confirm that the following items are present in the figure legend, table legend, main text, or Methods section.

n/a	Confirmed	
☐	☒	The exact sample size (n) for each experimental group/condition, given as a discrete number and unit of measurement
☐	☒	A statement on whether measurements were taken from distinct samples or whether the same sample was measured repeatedly
☐	☒	The statistical test(s) used AND whether they are one- or two-sided<br>*Only common tests should be described solely by name; describe more complex techniques in the Methods section.*
☒	☐	A description of all covariates tested
☒	☐	A description of any assumptions or corrections, such as tests of normality and adjustment for multiple comparisons
☐	☒	A full description of the statistical parameters including central tendency (e.g. means) or other basic estimates (e.g. regression coefficient) AND variation (e.g. standard deviation) or associated estimates of uncertainty (e.g. confidence intervals)
☐	☒	For null hypothesis testing, the test statistic (e.g. F, t, r) with confidence intervals, effect sizes, degrees of freedom and P value noted<br>*Give P values as exact values whenever suitable.*
☒	☐	For Bayesian analysis, information on the choice of priors and Markov chain Monte Carlo settings
☒	☐	For hierarchical and complex designs, identification of the appropriate level for tests and full reporting of outcomes
☒	☐	Estimates of effect sizes (e.g. Cohen's d, Pearson's r), indicating how they were calculated

Our web collection on statistics for biologists contains articles on many of the points above.

Software and code

Policy information about availability of computer code

Data collection	no specific software was used
Data analysis	RNA sequencing reads were mapped to the reference human genome assembly of Ensembl GRCh37 annotation version 75 using STAR with default parameters. Reads mapped to each gene were counted using FeatureCount and transformed to RPKM. STAR-fusion was utilized to identify and annotate gene fusion candidates, using the fastq files as input. For Whole gnome sequencing Sequencing reads were then cleaned and mapped to hg19 reference genome using bwa mem. Duplicates were marked using Picard MarkDuplicates tool (https://broadinstitute.github.io/picard/). Structural variants were identified using Manta and the variant related to the MGMT fusion was manually picked.<br>The 1p/19q codeletion status was predicted using the custom CNAPE software available at https://github.com/WangLabHKUST/CNAPE.<br>Data analysis was also performed with the R programming language (3.4).<br>Software versions:<br>samtools 1.2, picard MarkDuplicates 2.9.2, STAR 2.6.1d, featureCounts 1.5.1, STAR-Fusion 1.5.0, bwa 0.7.15-r1140, manta 1.4.0, R 3.5.3, ComplexHeatmap 1.2.0, survival 2.44-1.1, ggplot2 3.2.1, survminer 0.4.6., FlowJo 9.9.4, CytoVision Version 7.4, GraphPad Prism 6

For manuscripts utilizing custom algorithms or software that are central to the research but not yet described in published literature, software must be made available to editors and reviewers. We strongly encourage code deposition in a community repository (e.g. GitHub). See the Nature Research guidelines for submitting code & software for further information.

Data

Policy information about availability of data

All manuscripts must include a data availability statement. This statement should provide the following information, where applicable:

- Accession codes, unique identifiers, or web links for publicly available datasets
- A list of figures that have associated raw data
- A description of any restrictions on data availability

The raw sequencing data of the newly sequenced samples are deposited in the Genome Sequence Archive in BIG Data Center, Beijing Institute of Genomics (BIG), Chinese Academy of Sciences, under accession number BioProject ID: PRJCA001580 that are publicly accessible at https://bigd.big.ac.cn/bioproject/browse/PRJCA001580. Data from SMC were available in EGA (https://www.ebi.ac.uk/ega/datasets/EGAS00001001800), data from TCGA were downloaded from NCI Genomics Data Commons (GDC) data portal (https://portal.gdc.cancer.gov). Previously published CGGA data have been uploaded to the Genome Sequence Archive in BIG Data Center, Beijing Institute of Genomics (BIG), Chinese Academy of Sciences, under accession number BioProject ID: PRJCA001746 (https://bigd.big.ac.cn/bioproject/browse/PRJCA001746) and PRJCA001747 (https://bigd.big.ac.cn/bioproject/browse/PRJCA001747). The reference human genome hg19 is downloaded from http://hgdownload.cse.ucsc.edu/goldenpath/hg19/bigZips/hg19.fa.gz, while the genome annotation file is downloaded from ftp://ftp.ensembl.org/pub/release-75/gtf/homo_sapiens/Homo_sapiens.GRCh37.75.gtf.gz. All the other data supporting the findings of this study are available within the article and its information files and from the corresponding author upon reasonable request.
Figures with source data availabe: 1a, 1d-e, 2b, 2c, 2d, 3c-d, 3e-f, 4a, 4d, 4e, 4f, S1a-f S7e

Field-specific reporting

Please select the one below that is the best fit for your research. If you are not sure, read the appropriate sections before making your selection.

☒ Life sciences ☐ Behavioural & social sciences ☐ Ecological, evolutionary & environmental sciences

For a reference copy of the document with all sections, see nature.com/documents/nr-reporting-summary-flat.pdf

Life sciences study design

All studies must disclose on these points even when the disclosure is negative.

Sample size	No sample size calculation was performed. Number of experimental animals per group was chosen based on previous experiments using similar experimental settings.
Data exclusions	No data were excluded from the analysis
Replication	Experiments were performed with three different biological replicates and at least two technical replicates. All attempts at replication were successful.
Randomization	For the xenograft U251 model, animal were randomized in two groups based on BLI output. For the xenograft h543 model animal were randomized in two groups with similar number of males and females
Blinding	Investigators were not blinded for group allocation in the xenograft experiments. Blinding was not possible since TMZ treated was performed by only one person.

Reporting for specific materials, systems and methods

We require information from authors about some types of materials, experimental systems and methods used in many studies. Here, indicate whether each material, system or method listed is relevant to your study. If you are not sure if a list item applies to your research, read the appropriate section before selecting a response.

Materials & experimental systems

n/a	Involved in the study	
☐	☒	Antibodies
☐	☒	Eukaryotic cell lines
☒	☐	Palaeontology and archaeology
☐	☒	Animals and other organisms
☐	☒	Human research participants
☐	☒	Clinical data
☒	☐	Dual use research of concern

Methods

n/a	Involved in the study	
☒	☐	ChIP-seq
☐	☒	Flow cytometry
☒	☐	MRI-based neuroimaging

Antibodies

Antibodies used	MGMT (Biosciences, Cat. 557045, Lot. 6280927, 1:2000),<br>Alix (Cell Signaling, Cat. 2171, Lot. 5, 1:1000)<br>TSG101 (BD Transduction Laboratories, Cat. 612696, Lot. 7208980, 1:2000)<br>p85 (Millipore, Cat. 0619, Lot. 3009962, 1:10000)<br>GAPDH (Santa Cruz, Cat. Sc-365062, Lot. J1314, 1:500)<br>Vinculin (Sigma-Aldrich, Cat. V9131, 1:10.000)<br>H2AX (Ser139) (pH2AX, JBW301, Millipore, Cat. 05-636, Lot. DAM1493341, 1:4000)<br>H2AX (Ser139) (pH2AX, Merck, Cat. 05-363, Lot. 2310355, 1:1000)<br>53BP1 (Novus Biologicals, Cat. NB100-304, Lot. A2, 1:3000)<br>anti-BrdU (BU-1;GE Healthcare, RPN202, Lot. 341585, 1:100)<br>Alexa 488 (rabbit) (Life Technologies, Cat. A-21206, Lot. 198155)<br>Alexa 555 (mouse) (Life Technologies, Cat. A-31570, Lot. 1048568)
Validation	MGMT: Immunoprecipitation (IP) and Western Blot analysis (WB) of MGMT in MOLT-4 cells . MOLT-4 (Human T-lymphoblasts; ATCC CRL-1582) cell lysates were immunoprecipitated with the Mouse Anti-Human MGMT antibody (lane 1) or with an isotype control (lane 2). MGMT is identified as an ~25 kD band.<br>Alix: Nature Communications on 29 April 2020 by Sung, B. H., von Lersner, A., et al.; Journal of Extracellular Vesicles on 15 April 2020 by Dhondt, B., Geeurickx, E., et al..<br>TSG101: Western blot analysis of TSG101 on a K-562 cell lysate (Human bone marrow myelogenous leukemia; ATCC CCL-243)<br>p85: Nature Communications on 13 April 2018 by Oldrini B. , Curiel, A., et al.<br>GAPDH: Western blot analysis of GAPDH expression in F9, EOC 20 and C6 whole cell lysates and rat testis tissue extract<br>Vinculin: Minamishima YA, et al. Blood 111(6), 3236-3244, (2008); Hama T, et al. Oncologist 14(9), 900-908, (2009)<br>pH2AX (JBW301): 2 µg/ml of this antibody detected phosphorylated histone H2A.X in HeLa cells treated with 0.5 µM staurosporine for 4-6 hours.<br>pH2AX (Merck): Immunocytochemistry,Enhanced Validation-Recombinant Antibody Technology.<br>Immunofluorescent analysis of HeLa cells was performed using a 1:100 dilution of Cat. No. ZRB05636, Anti-phospho-Histone H2A.X (Ser139), clone 6L16 ZooMAb® Rabbit Monoclonal and visualized with a Goat Anti-Rabbit secondary antibody conjugated to Alexa Fluor® 488 (Green). Actin filaments have been labeled with phalloidin (Red). Nucleus is stained with DAPI (Blue). This antibody positively stains the nucleus.<br>53BP1: Knockout Validated: 53BP1 Antibody [NB100-304] - 53BP1 was detected in immersion fixed HeLa cells (left) but was not detected in 53BP1 knockout Hela cells (right) using Rabbit Anti-human 53BP1 polyclonal antibody (Catalog #NB100-304) at 0.3 ug/mL for 3 hours at room temperature. Cells were stained using the NorthernLights™ 557-conjugated Anti-Rabbit IgG Secondary Antibody (red; Catalog # NL004) and counterstained with DAPI (blue). Specific staining was localized to nuclei.<br>BrdU: Cell Death & Disease on 14 April 2020 by Jiang, K., Yao, G., et al.; Genes on 11 January 2020 by Lantz, B., White, C., et al..

Eukaryotic cell lines

Policy information about cell lines

Cell line source(s)	The human glioma cell lines U251 (Sigma-Aldrich, 09063001) was kindly provided by Eric Holland and U87 (HTB-14) was purchased from ATCC. The Gp2-293 packaging cell line was purchased from Clontech (Cat. 631458).
Authentication	DNA fingerprinting has been performed for authentication of the U251 and U87 cell lines (data available upon request). Gp2-293 were not authenticated.
Mycoplasma contamination	All the cell lines were Mycoplasma negative and routinely checked for contamination by PCR analysis.
Commonly misidentified lines<br>(See ICLAC register)	No commonly misidentified cell lines were used in the study.

Animals and other organisms

Policy information about studies involving animals; ARRIVE guidelines recommended for reporting animal research

Laboratory animals	Immunocompromised nu/nu 6-8 weeks old mice. Mice were housed in the specific pathogen-free animal house of the Spanish National Cancer Centre under conditions in accordance with the recommendations of the Federation of European Laboratory Animal Science Associations (FELASA).
Wild animals	The study didn't involve wild animals
Field-collected samples	The study didn't involve samples collected in the field
Ethics oversight	All animal experiments were approved by the Ethical Committee (CEIyBA) and performed in accordance with the guidelines stated in the International Guiding Principles for Biomedical Research Involving Animals, developed by the Council for International Organizations of Medical Sciences (CIOMS).

Note that full information on the approval of the study protocol must also be provided in the manuscript.

Human research participants

Policy information about studies involving human research participants

Population characteristics	To reveal the landscape of TMZ resistance in glioma patients, we analyzed RNA-sequencing data of 252 TMZ-treated recurrent gliomas, among which 105 (42%) were newly collected. 154 (61%) Males and 98 (39%) Females; 216 (85.7) Asian and 36 (14.3%) Non-Asian; 17 (6.7%) Grade II, 38 (15.1%)Grade III and 197 (78.2%)Grade IV.
Recruitment	The newly sequenced tumor were collected from Beijing Tiatan Hospital as part of the Chines Glioma Genome Atlas (CGGA, http://cgga.org.cn/).
Ethics oversight	The study was approved by the institutional review board in Capital Medical University (IRB ID: KYSB2015-23). Informed consent was obtained from each patient before surgery.

Note that full information on the approval of the study protocol must also be provided in the manuscript.

Clinical data

Policy information about clinical studies

All manuscripts should comply with the ICMJE guidelines for publication of clinical research and a completed CONSORT checklist must be included with all submissions.

Clinical trial registration	Not applicable.
Study protocol	Not applicable.
Data collection	The newly sequenced tumor were collected from Beijing Tiatan Hospital as part of the Chines Glioma Genome Atlas (CGGA, http://cgga.org.cn/).
Outcomes	Not applicable.

Flow Cytometry

Plots

Confirm that:

☒ The axis labels state the marker and fluorochrome used (e.g. CD4-FITC).

☒ The axis scales are clearly visible. Include numbers along axes only for bottom left plot of group (a 'group' is an analysis of identical markers).

☐ All plots are contour plots with outliers or pseudocolor plots.

☐ A numerical value for number of cells or percentage (with statistics) is provided.

Methodology

Sample preparation	Cells were seeded in 6-well culture plates (100,000 per well) in duplicates and cultured in presence of Temozolomide (100uM) and/or O6-Benzylguanine (100uM) for 72 hours. Cells were then harvested by phosphate-buffered saline (PBS), washed twice in cold PBS, fixed with cold 100% Ethanol on ice for 30 minutes and pelleted by centrifugation at 1200 rpm for 10 minutes. Pellet was then washed twice with PBS and 1% fetal bovine serum (FBS) and stained with 200ul of propidium iodide (PI) (50ug/ml) overnight.
Instrument	Samples were acquired on a FACS Canto II (Beckton Dickinson).
Software	All data were analyzed using FlowJo 9.9.4 (Treestar, Oregon)
Cell population abundance	60-80%
Gating strategy	585_42-Blue-A :: PI/585_42-Blue-H :: PI to select single cells; FSC-A/SSC-A to exclude debris. See Supplementary Figure 8 for gating strategy.

☒ Tick this box to confirm that a figure exemplifying the gating strategy is provided in the Supplementary Information.
